# Supplementary material for: Plant Robots: Harnessing Growth Actuation of Plants for Locomotion and Object Manipulation
Source: Adv Sci (Weinh). 2024 Sep 23;11(43):2405549. doi: 10.1002/advs.202405549 (PMC11578339; doi:10.1002/advs.202405549)
Supplement: Supplementary file 1 — Supporting Information [file ADVS-11-2405549-s003.docx]

Supporting Information

Plant robots: harnessing growth actuation of plants for locomotion and object manipulation

K. Murakami, M. Sato, M. Kubota, J. Shintake*

Holding force measurement of the gripper

**
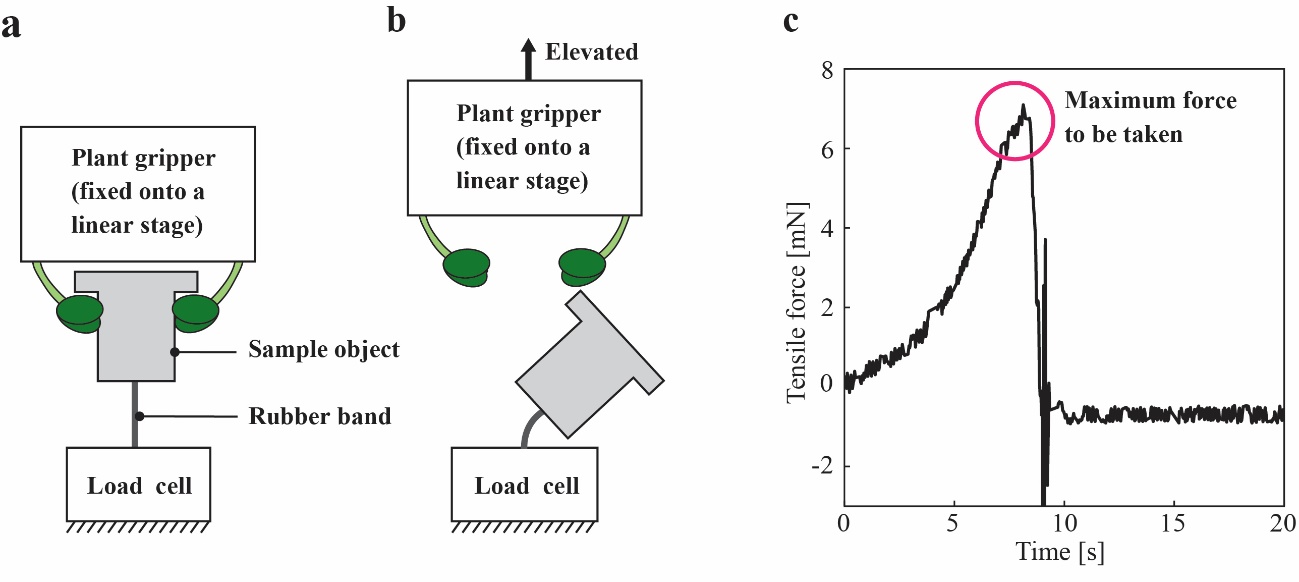
**

**Figure S1.** (a) Schematic of the gripper holding the sample object, mounted onto a linear motorized stage. The lower part of the sample object is connected to a load cell, which is fixed on the ground, using a thin rubber band. (b) Schematic depicting the elevated gripper. Under this condition, the tensile force acting on the load cell is measured until the sample object detaches from the gripper. (c) Example of measured force data.
